# Mobile Device Use Among Rural, Low-Income Families and the Feasibility of an App to Encourage Preschoolers’ Physical Activity: Qualitative Study

**DOI:** 10.2196/10858

**Published:** 2018-12-06

**Authors:** Morgan L McCloskey, Darcy A Thompson, Barbara Chamberlin, Lauren Clark, Susan L Johnson, Laura L Bellows

**Affiliations:** 1 Department of Food Science and Human Nutrition Colorado State University Fort Collins, CO United States; 2 Department of Pediatrics University of Colorado Anschutz Medical Campus Aurora, CO United States; 3 Media Productions and Learning Games Lab New Mexico State University Las Cruces, NM United States; 4 College of Nursing University of Utah Salt Lake City, UT United States

**Keywords:** smartphone, mobile apps, families, child, preschool, physical activity, rural population, poverty

## Abstract

**Background:**

As mobile devices are becoming ubiquitous, technology-based interventions provide a promising strategy to positively influence health behaviors of families with young children. However, questions remain about the feasibility and acceptability of intervention delivery via mobile apps in low-income, rural settings and among families with preschoolers.

**Objective:**

The aims of this study were to understand the content and context of mobile device use for preschoolers; explore parent beliefs on this topic, including the acceptability of intervention delivery via mobile devices; and test a prototype of an app to encourage preschoolers’ physical activity with both parents and children.

**Methods:**

Parents (n=29) were recruited from 5 preschool centers in eastern, rural Colorado to complete a semistructured telephone interview regarding preschoolers’ mobile device use. A second sample of parents (n=31) was recruited from the same preschool centers to view the app prototype independently and provide feedback. A third sample of preschool children (n=24) was videotaped using the app in small groups to measure engagement and record their responses to the app.

**Results:**

Five key content areas emerged from the telephone interviews: (1) mobile devices are an important part of families’ everyday routines, and parents have parameters governing their use; (2) parents often use mobile devices as a tool for behavior management; (3) parents clearly distinguish between mobile device use for learning versus entertainment; (4) parents have an overarching desire for balance in regard to their child’s mobile device use; and (5) parents were generally supportive of the idea of using mobile apps for intervention delivery. From the app prototype testing with parents, participants reacted positively to the app and felt that it would be useful in a variety of situations. Testing with preschoolers showed the children were highly engaged with the app and a majority remained standing and/or actively moving through the entire length of the app.

**Conclusions:**

Mobile devices are already integrated into most families’ daily routines and appear to be an acceptable method of intervention delivery in low-income families in rural Colorado. The physical activity app represents an innovative way to reach these families and, with further improvements based on participant feedback, will provide children with a unique opportunity to practice key movement skills.

## Introduction

The use of electronic multimedia (eg, educational games, mobile apps, and personalized electronic messages) in interventions has tremendous potential to improve the health behaviors and knowledge of children and their parents, including activity behaviors. Several advantages exist in using mobile health (mHealth) modalities to deliver childhood obesity interventions, including reduced participant burden, ability to deliver more exciting and visually appealing messages, novel content formats such as mobile games, and greater flexibility [1]. To date, one of the few studies using a parent-focused mHealth intervention to target early childhood audiences reported sustained parental engagement with the smartphone app and improvement in certain diet and physical activity behaviors [2]. Among a wider age range of children, evidence suggests that electronic interventions on obesity can positively affect dietary and physical activity behaviors [3,4].

Interventions focused on early childhood are promising, in part, because early habits can translate into adolescence and even adulthood, making early childhood a crucial time to establish healthy habits [5]. Not only are children influenced by the physical and social environment of the home [6] but also parental behaviors related to physical activity have been shown to be associated with their preschool child’s health behaviors [7]. Outside of preschool, young children spend a majority of their time at home, making parents and the home and family environment a strong intervention target for improving preschool children’s activity behaviors.

To address these behaviors and positively influence the home environment, there is a need for novel strategies, particularly in low-resource families who may face time and access challenges to participating in educational or intervention programs [8]. With the continued rise in mobile device ownership and use across all populations, electronic formats are a realistic and desirable way to engage these families [9,10]. A recent survey by Common Sense Media found that mobile devices are nearly ubiquitous in the homes of families with young children, as 98% of children younger than 8 years live in a home with some type of mobile device, and children spend about 48 min a day using a mobile device [11]. These data are echoed by a variety of other surveys [12-15], including a previous study of lower-income families with preschoolers targeted toward rural, northeastern Colorado, which found that 91.6% (175/191) of preschoolers had access to mobile devices [16].

In addition to information on usage, parents’ views about preschooler use of mobile devices have also begun to be investigated. The Common Sense Media survey reported that parents of children in the age group of 0 to 8 years have rather mixed views of children’s media use. A majority of parents (76%) believe that *the less time kids spend with screen media, the better off they are*, yet 74% also believe that their *child benefits from the screen media he/she uses* [11]. With regard to specific benefits of screen media, 75% of parents of children aged between 3 and 5 years believe that media help their child with learning, and 62% believe it helps their child with creativity [11]. Other studies have reported similar tensions around parental beliefs related to mobile devices, with many parents simultaneously recognizing the potential benefits of mobile devices (such as children’s learning or entertainment) and expressing concerns about potential drawbacks to mobile device use (such as exposure to inappropriate content or lack of engagement with others) [17-19]. However, beyond these overarching views of mobile devices, the specific role of mobile devices in the daily lives of families, as well as the acceptability of using mobile devices for intervention delivery in low-resource populations, remains unknown. Therefore, before moving forward with an intervention, formative research to understand the feasibility of mobile devices as a mode of intervention delivery in rural, low-resource audiences is warranted.

In addition to feasibility, it is critical to pretest intervention materials, such as prototypes of mobile device apps, to determine which concepts and materials resonate best with the target audience. The goal of pretesting messages and materials is to assess message appeal, recall and comprehension, sources of confusion or offense, and motivation to act. Siegel notes that pretesting materials will not tell researchers exactly how materials will perform, and they will identify any *red flags* in terms of unintended interpretations and executional details that need changing (colors, music, voices, timing, etc) [20]. Pretesting ensures that the final versions of materials contain messages that are clear, effective, true to the strategy, and easily understood by the intended audience and that they do not generate unintended reactions [20].

This paper presents results associated with one component of a larger, mixed-methods, formative research study designed to inform a technology-based interactive family intervention focused on young children’s physical activity and eating behaviors [21]. The aims of the study component presented in this paper were to (1) understand the content and context of preschoolers’ mobile device use as well as parent beliefs and values on this topic, including an exploration of the acceptability of intervention delivery via mobile devices and (2) pretest an initial prototype of an app to encourage preschoolers’ physical activity with both parents and children.

## Methods

### Study Design

This study focused on families living in rural areas of Colorado who had a child attending Head Start or Colorado Preschool programs (ie, federally or state-supported preschool programming). For the first study aim related to mobile device use, content, and context, semistructured telephone interviews were conducted with parents of preschoolers. To address the second study aim of prototype pretesting, face-to-face interviews with parents of preschoolers were conducted to gather feedback and reactions to the prototype, which were then followed by pretesting with preschoolers. This paper sequentially presents the results associated with the (1) telephone interviews with parents; (2) prototype pretesting with parents; and (3) prototype pretesting with preschoolers. It should be noted, however, that although presented sequentially, the findings related to general mobile device use did not inform design of the physical activity app. The study was approved by Colorado State University’s Institutional Review Board.

### Participants

#### Telephone Interviews

Parents were recruited from 5 Head Start and preschool centers in eastern, rural Colorado. Parent packets, including an informed consent and phone interview interest form, were sent home in children’s backpacks in the spring of 2016. Parents had 2 to 3 weeks to return the interest form; preschool center staff were engaged in the process to encourage families to participate. In total, 110 participants returned a form, expressing interest in the phone interview. To ensure representation across sites and demographic groups, participants were stratified by site, parental education level, and ethnicity. A total of 73 participants were then systematically chosen to contact from within these groups, and 29 completed the phone interview. All those completing the phone interview received US $20.

#### Prototype Pretesting

##### Parents

In the spring of 2017, parent packets were sent home in children’s backpacks at the same 5 Head Start and preschool centers to recruit additional parents to gather reactions on a prototype of an app to encourage physical activity among preschoolers. Parents again had 2 to 3 weeks to return the interest form; a total of 102 participants returned the form. Participants were stratified by site and child gender; 60 were systematically chosen from these groups to contact, and 32 completed the interview and were compensated US $40 for their time.

##### Preschoolers

Preschoolers were recruited from a university-affiliated preschool that serves families from a full range of income levels. To engage preschoolers to test the app prototype, an informational sheet was distributed to parents, who were given 2 weeks to opt out of participation for their child. Only 1 family opted out of participation. All other children at the preschool who were between the ages of 3 and 5 years and were present on at least one of the pretesting days participated (n=24).

### Study Procedures

#### Telephone Interviews

Semistructured telephone interviews were conducted with parents to understand the role of mobile devices in their families’ daily life. The semistructured interview schedule consisted of 25 questions with probes and was designed to produce comparable, descriptive information about model device use [22]. Key topics included child mobile device use, typical household practices related to mobile device use, and parent values and beliefs related to mobile device use. At the end of the semistructured telephone interview with parents, participants were also asked for their opinions regarding the feasibility of hypothetical intervention components, including an app to encourage physical activity. Sample questions are presented in Table 1.

Experts in child development, public health, technology and instructional design, medical anthropology, and pediatrics reviewed the interview script, and it was piloted with 2 members of the target audience to finalize the order, flow, and wording of questions. The interviews were conducted by 2 research associates, who had each been trained in qualitative research and interview best practices [23]. Interviews ranged between 20 and 35 min, and all were audio-recorded with participants’ permission. Preliminary analysis of the telephone interviews included member checking, in which key points and interpretations from each interview were summarized and sent to the interview participants for feedback.

#### Prototype Pretesting

##### Parents

As part of a longer interview with parents about their preschooler’s physical activity (data reported elsewhere), a 5-min prototype of a mobile app to encourage physical activity in preschoolers, *Jungle Gym*, was shown to parents to gather their feedback. Parents viewed the app without their children and were asked to anticipate how it could be used. They were also asked their general thoughts about the app, what situations they could envision their preschooler using the app, and how they would use the app with their child.

**Figure 1:**
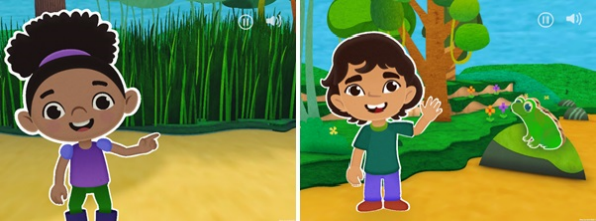
Screenshots of mobile app prototype to encourage physical activity among preschoolers.

The app is intended to enhance language related to movement, help children practice gross motor skills, and provide an opportunity for parents and children to interact together related to movement if they so desire. Although mobile devices are often perceived as sedentary devices, the development team sought to create an app that would facilitate movement in young children and model the kinds of off-device play activities that would also make children more active. The app begins when an animated character invites the user to go on a jungle adventure and states, “we will be moving our bodies, so make sure you have plenty of space.” After this introduction, the child can set the iPad on a surface and go through a series of short adventures, each highlighting a specific motor skill: running, jumping, leaping, hopping, galloping, or side-stepping. For example, users are asked to run quickly through the jungle to escape rain, jump to harvest a mango from a tree, or hop alongside a frog (Figure 1). The short adventures or *scenes* are presented to the children in a random order each time the app is played in an effort to sustain children’s interest in engaging with the app.

**Table 1:** Sample questions asked in semistructured telephone interviews with parents about mobile device use, context, and content.

Overarching question	Probes
If you think about the last day or so, when did your child use a smartphone or tablet throughout the day?	Where do they use the mobile device? (inside the home, outside the home); Was this a typical day?; How did this use compare to a weekend day?
What are the main factors influencing when or how long your child uses a smartphone or tablet?	Does it depend on any of the following: who is supervising them, family schedule, weather?
What are some benefits of your child using a smartphone or tablet?	What are the benefits to you?; What are the benefits to your child?
Some parents have rules about how their child can use a smartphone or tablet. Other families do not. Do you or your family have any rules about how your child can use the smartphone or tablet?	If so, how or why did you come up with this rule?; If so, how well do these rules work?
Please tell me if there are any times or reasons that you encourage your child to use a smartphone or tablet?	Are there any specific places, situations, or times during the day that you encourage them to use a mobile device?; How easy or hard is it to encourage your child to use a mobile device?
Please tell me if there are any times or reasons that you discourage your child to use a smartphone or tablet?	Are there any specific places, situations, or times during the day that you discourage them to use a mobile device?; How easy or hard is it to discourage your child to use a mobile device?

##### Preschoolers

The app prototype described above was further refined based on parental feedback and was then tested with 24 preschoolers to understand children’s initial reactions to the app. Children were taken out of their classroom in groups of 3 to the gym room of the preschool by 2 members of the research team. The app was introduced to the children, including a brief explanation that they would be asked to stand up and move along with the characters in the app. The 4-min app prototype was shown to each group of children 1 or 2 times (children were allowed to go through the app a second time if they asked to do so). After using the app, children were asked if they enjoyed playing the app, as well as what they liked and did not like about the app. In total, the activity lasted about 10 to 15 min per group, and children’s use of the apps and reactions were recorded via videotape and researcher notes. In this session, children used the app in small groups, without their parents.

### Data Analysis

#### Telephone Interviews

After sending summaries to participants for member checking, no additional information was provided. Using best practices for reproducibility in qualitative data preparation [24], all interviews were deidentified and transcribed verbatim by a Health Insurance Portability and Accountability Act–compliant vendor, verified for accuracy, and the verified transcripts imported into NVivo qualitative data analysis software (QSR International Pty Ltd Version 11, 2015). Two team members read the interview transcripts several times, and each created a preliminary codebook, including codes and definitions organized by overarching category. Three trained research team members coded 2 interview transcripts together to further refine code definitions and inclusion and exclusion criteria. Next, the 3 coders independently completed 1 set of coding with 3 transcripts, followed by a second set of 3 transcripts and established high interrater reliability (kappa=0.90 and 0.94, respectively). The remaining transcripts were split between 2 coders, with each reviewing the initial coding of another. Discrepancies were resolved through discussions with the third coder. Summary reports were generated by code, and thematic analysis was used to analyze the results [25]. Two researchers independently read through all the quotes associated with each code, and each identified emerging themes. The researchers met several times to achieve consensus and further refined each theme in the context of both the original codes and entire dataset.

#### Prototype Pretesting

##### Parents

Parent’s reactions to the app prototype were also recorded and transcribed verbatim. A general inductive approach was used [26], in which the responses were analyzed by 2 researchers who independently read transcripts multiple times, discussed the responses to reach consensus, and generated a summary of participant responses.

##### Preschoolers

The research team reviewed the notes and videotapes of the children using the app to understand whether the app represented a feasible approach to engage children. General reactions and engagement level, such as asking to play the app again and statements of what kids liked and did not like about the app, were compiled. Two researchers watched the videos multiple times to provide a general estimate of the percentage of time each child spent engaged in the following 4 categories: moving along with the characters as directed, engaging in other active and creative movements, becoming distracted by something else in the gym room, or no movement (sitting down).

## Results

### Participant Characteristics

In the telephone interviews addressing mobile device use, content, and context, 93% (27/29) of the participants (n=29) were mothers, 41% (12/29) identified as Hispanic, and 66% (19/29) represented households making less than US $49,999 per year. Phone interview participants represented a wide range of education levels, with 45% (13/29) of participants having a high school diploma or less. In the prototype pretesting phase with parents (n=31), 77% (24/31) of participants were mothers, 36% (11/31) identified as Hispanic, 62% (19/31) represented households making less than US $44,955 per year, and 33% (10/31) of participants had a high school diploma or less. Only1 participant completed both interviews. The preschoolers who tested the physical activity app were aged 3 years (n=1) and 4 years (n=23), and 50% (12/24) were male.

### Telephone Interviews

Five key themes emerged from the telephone interviews: (1) mobile devices are an important part of families’ everyday *routines*, and parents have parameters governing their use; (2) parents often use mobile devices as a tool for *behavior management*; (3) parents clearly distinguish between mobile device use for *learning versus entertainment*; (4) parents have an overarching desire for *balance* with regard to their child’s mobile device use; and (5) parents were generally *supportive* of the idea of using mobile apps for intervention delivery.

#### Routines and Family Guidelines

Mobile devices have become a part of family routines, as a majority of parents indicated that their child used a smartphone and/or tablet daily or almost daily. All families owned at least one mobile device, and nearly half of children had their own device. Children used mobile devices for both learning and entertainment purposes, such as watching videos (YouTube for kids was particularly popular) or playing games based on cartoons and other characters popularized through television. Families varied in when the child used mobile devices, and many had strong feelings as to when the child could or could not engage with the device (ie, in the morning before school or before bedtime or while parents needed the child to be occupied).

Some parents co-used mobile devices with children most commonly to show a child how to use an app for the first time, to take turns playing a game, or to help them learn something. Many parents valued it as time spent together, particularly when it meant *cuddling up* or *sitting really close together*. However, other families mentioned that they played together in other ways and viewed time with mobile devices as an alone activity or *alone time*.

Parents had a variety of parameters in place to manage their child’s use of mobile devices, particularly related to content and timing of use. To control the content that their preschooler accessed, parents used parental locks or child modes, monitored which apps their children used, had specific *kid areas* on devices, or simply engaged in conversations with the child to convey what children were allowed to use. No matter the strategy used, the primary concern of most parents was that their preschooler would see something *inappropriate*, followed by a concern that something would *get messed up* on the device.

Other important guidelines were related to the overall time children spent using devices. Whether an enforced rule or a more theoretical ideology, many parents mentioned that children were allowed to use devices only for a certain amount of time each day, particularly in the home setting. These time limits were usually set because parents did not want children to be *glued to a screen* all day and wanted them to get a variety of experiences in childhood, ranging from playing outside and getting dirty, developing social skills through interaction with other children and adults, and using their imagination:

I do try limiting it as much as I can. I’d rather them be outside playing and being kids.

Most participants had family-specific guidelines in place related to the situations in which their child was allowed to use the device. For example, policies on use before bedtime was mixed, as some families did not allow device use close to bedtime, whereas other families encouraged it at this time to help the child unwind. However, there was a consensus among parents that children should not use mobile devices during mealtime:

At dinnertime, we have no iPads or tablets on the table. You know that’s family time.

A large number of parents also did not allow preschoolers to use devices while spending time with extended family and/or friends, as parents preferred them to be socializing and interacting:

When we’re with family we try and have him not just be buried in his tablet and be part of the whole family together.

#### Behavior Management

Parents also commonly used mobile devices for behavior management. Nearly every parent felt that mobile devices were helpful to occupy children so that the parent had time to get something done, such as an important phone call, finishing work, household chores, or fixing dinner:

Oh, well, play this game here quietly so I can get this done.

Many parents also used mobile devices to keep their children entertained and quiet outside the home. Parents frequently mentioned that their child used mobile devices on long car rides when the family had an unexpectedly long wait in line, doctor’s appointments, or restaurants. There was a particular focus on using mobile devices to prevent preschoolers from *screaming* or *running around and going crazy* in these kinds of public settings:

It’s really nice to have to keep him entertained and keeps him from melting down in the restaurant, or in the doctor’s office or somewhere.

Some parents did express guilt at using devices in this way, but almost all seemed to have accepted it as a useful, and occasionally necessary, way to manage public outings. One mother summed it up well:

I was always that parent before who was like, “No. My kid will never play on electronic devices.” Then you take a five-year-old out in public, and then they throw a fit, and you’re just like, “Here. Take it.”

Beyond serving as an entertainment tool in public or when a parent is occupied, mobile devices were used strategically (eg, as rewards and consequences):

It gives me leverage with him. It’s his currency.

This quote reflects the sentiment of many parents: mobile devices are an effective tool parents use to manage behavior. Smartphone or tablet time could be taken away for refusing to share with a sibling, doing something inappropriate on the device, or even for poor behavior unrelated to mobile devices, such as *throwing a fit*, acting up, or not listening. On the other end of the spectrum, extra smartphone or tablet time was frequently offered as a reward for positive behavior, and many children had to earn their time on mobile devices by doing chores, such as picking up their clothes or toys. It is worth noting that a small group of families did not see mobile devices as a reward or consequence for their children. These families fall into 2 categories: those whose children do not use mobile devices frequently enough for it to be an effective reward and those families whose children exclusively use devices for learning, and thus taking it away would not be considered an appropriate strategy.

#### Learning Versus Entertainment

A clear difference in parent beliefs and practices emerged between children’s mobile device use for entertainment and learning. Nearly every parent mentioned that they wanted their child to learn from mobile devices. This learning fell into 3 main categories: learning how to use technology, in general, to be prepared for a technology-driven world; learning school readiness skills such as numbers, shapes and letters; or learning about a specific topic area that was of special interest to their child:

Of course technology is taking over the world, so it’s good that she is getting to learn how to use a tablet.

It’s beneficial in some learning areas...I want him to basically be prepared for kindergarten.

The last category of learning may be especially relevant for rural communities, as some parents mentioned technology as a way for children to understand more about the broader world outside their small town:

...just to gain background knowledge. We live in such a small town, we don’t necessarily get to see everything that maybe we would elsewhere.

Although a majority of parents mentioned learning as a benefit of mobile device use and wanted their children to be learning from devices, they also depended on the devices for entertainment in the behavior management situations mentioned above. Similarly, despite parents’ desires and values for children to use mobile devices for learning, most children used the devices for entertainment, both inside and outside the home. It should be noted, however, that children used different kinds of devices based on the activity: children were more likely to use smartphones for entertainment, whereas they used education-specific tablets (such as an Innotab, Nabi) for learning purposes. Furthermore, parents were more likely to co-use devices with their children when the use involved learning. Finally, parents tended to feel better about their children’s overall mobile device use when the child was learning from it, and many parents actually encouraged further use when educational content was being accessed:

I don’t think being on the phones or the tablets is a bad thing if they’re learning while they are on there.

Similarly, parents tended to be less strict with time limits when children were using educational content versus entertainment in the home:

I’m a little more lenient when he’s playing an educational game.

On the other hand, when devices were used for entertainment outside the home for behavior management, for example, waiting for appointments or at restaurants, many of the *rules* related to time limits did not seem to apply. Thus, mobile devices serve distinct purposes that are situationally dependent for children.

#### Balance

Overall, mobile device use is all about balance in these families. It is a balance between ensuring that children are learning while also keeping them entertained when the parent needs to get something done or the family has a long wait in a public setting. It is a balance between children understanding how to use mobile devices in a technology-driven world and children developing important social skills through off-screen interactions with friends and relatives. A quote from 1 mother clearly sums up these sentiments:

I really just didn’t want my children having their entire lives ruled by technology, but I didn’t want them to be so far out of the spectrum that they are typing with two fingers…enough to get them the knowledge base that they need without taking away the time that they spend with their friends and their family and outside.

#### Mobile Apps for Intervention Delivery

A majority of parents responded positively to the idea of using mobile apps, particularly if they were approved by the preschool center and helped their child to learn in some way. Most parents expressed that any mobile apps encouraged as part of a program through their preschool would simply replace existing mobile device activities, so the overall amount of time children spent on devices would not necessarily increase. In response to the idea of an app to encourage physical activity, parents were supportive, particularly if the app would help their preschooler to be active while using a device. A few parents did express doubts about the idea, as they felt that being active required putting down all devices and going outside to play:

You can’t learn how to ride a bike from a phone.

However, a majority of parents reacted positively to the idea, saying that it would be “cool,” “fun,” and “[their child] would love that.” Many parents felt that the app was something their child would actually use. Several mentioned the app would be especially helpful during the winter or other inclement weather to give children a structured way to get up and move around indoors:

I think that would be good for maybe wicked cold, snowy days or really rainy, funky days. It still gets them up and moving.

### Prototype Pretesting

#### Parents

Following the initial feedback, a prototype of the physical activity app was developed, and prototype pretesting was completed with parents to understand their reactions to an actual app prototype. Parents reacted positively to the app overall and thought it was a helpful way to combine mobile device use and activity. Parents were split on whether or not they would use the app with their child. Some parents felt that the app would be a good opportunity to engage with their child related to activity:

It might be something that I would try to use to do exercising and stuff together with him.

However, others expressed that they would use the app for behavior management, stating it would be helpful to *bring them down a little bit, while still keeping them active* or to entertain the child while the parent was getting chores done:

That would be a good one she can do while I’m doing laundry or something like that.

A few parents expressed concern about whether or not the app would be sufficiently engaging for their child. Several confirmed the opinions of previous parents that the app would be useful for encouraging child activity in the winter or on cold rainy days when it was not possible to be active outside. Other parents had even more creative ideas for using the app, ranging from lines at the grocery store to pit stops on road trips:

Anytime that there’s not enough space to do the full running activities, things like that. Car trips, probably, pit stops along road trips. It would be handy.

#### Preschoolers

Overall, the pretesting with preschoolers demonstrated that they were engaged by the app, and all children completed some physical activity as instructed by the app. When asked what they liked about the app, responses ranged from *the animals* to *it was fun*. When asked what they did not like about the app, a few children said it made them *tired*, indicating that they were putting a full effort into their movement. As they all asked to play more than once, all groups (n=9) of children used the app 2 consecutive times, and several groups expressed disappointment when they were not allowed to go through the adventure a third time. In 8 of 9 groupings, children remained standing and/or moving throughout the entire length of the app. In 6 groups, children were very active and were continuously engaged in either app-directed movements or were inspired to do other creative movement activities (running in circles, acting out animals, etc). Overall, there did not appear to be any difference in the level of engagement between the first and second round of use. Children’s reactions or confusion on specific interface issues were also recorded. A few of the children misunderstood directions related to more complicated motor skills, and it was observed that some children tended to move only when the character was talking, so the research team recommended that the voiceover script should be updated to increase clarity on directions for certain motor skills and increase repetitions to keep children on task and engaged as much as possible with each activity. Additionally, a moving figure will be added to the app to demonstrate all movement skills. Additional recommendations made to the developers included improvements to the storyline, including more animals to meet on the jungle adventure and silly noises. These recommendations will be incorporated in future versions of the app.

## Discussion

### Principal Findings

The overarching goals of this study were to understand mobile device use in the target audience and pretest a prototype of an app to promote physical activity among preschoolers as 1 piece of a larger formative research study designed to inform a technology-based interactive family nutrition and physical activity intervention. The first component of this study deepened the understanding of preschoolers’ use of mobile devices, the role that mobile devices play in the daily lives of lower-income rural Colorado families and demonstrated the acceptability of intervention delivery via a mobile platform. The second component confirmed the feasibility of the app and gathered crucial feedback from parents and children, which will be used to inform improvements in the app moving forward.

#### Telephone Interviews

Mobile devices are an important part of everyday routines in these families, echoing other findings that preschoolers are frequent users of mobile devices [12-15]. In contrast to the Common Sense Media Survey 2013, which found disparities in tablet ownership by family income level [12], every family interviewed had multiple mobile devices, and about half of preschoolers actually had their own mobile device. However, there was some evidence of the disparities in access to educational content among lower-income families, as identified by Common Sense Media 2013 [12]. Many parents in our sample mentioned that they were not able to afford any sort of paid educational content, which could potentially be associated with limits on the quality of educational materials that low-income children are accessing.

Beyond the incorporation of mobile devices into family routines, another clear content area that emerged was the use of mobile devices for behavior management, reflecting findings from several other studies [17,18,27,28]. Even before the onset of mobile devices, Rideout et al found that parents commonly used media *to keep their kids occupied while they [parents] get chores done* [17]. In 2010, Chiong and Shuler observed the *pass-back* effect in which parents would deliberately hand over their own smartphone or tablet to their young child to keep them occupied and entertained in a public setting [27]. More recently, 2 other qualitative studies, encompassing families from a variety of locations and socioeconomic backgrounds, also found that parents used mobile devices to keep children quiet or entertained when the parent needed undisrupted time to get something done [18,28].

A final content area was the clear distinction between the use of devices for learning versus entertainment and the desire for balance in children’s mobile device use. In the home, parents strongly preferred educational content and would even relax the rules about time limits or encourage additional use when they felt their child was learning. However, if the child was watching a video or playing a noneducational game at home, time limits were more strictly enforced, and children were encouraged to do other activities. Outside the home, when parents were using mobile devices as a tool for behavior management, they simply wanted to keep their child entertained and did not have a strong preference as to the content that they were accessing. Thus, parental values and beliefs may be conditional, whether they are conscious of it or not.

Other studies have found a similar desire for balance related to mobile device use in families with young children [17-19]. For example, Radesky et al also found that parents expressed tension between the potential educational benefits of mobile devices and the potential negative effects on social skill development [18]. These parents similarly struggled with the usefulness of devices in keeping children entertained and quiet versus missing quality family time [18]. Another report on media use with young children found that parents appreciated that media allowed for *me* time and the ability to get things done and also exposed their child to a variety of new learning experiences [17]. |Finally, a survey of parents of children 6 months to 5 years of age found that the top 3 parental motivations for child media use were the child’s enjoyment of the media, the educational value of media, and media use so that parents can accomplish their own chores, again indicating the importance of balance in mobile device use [19].

#### Prototype Pretesting

Additionally, this study demonstrated the feasibility of a mobile app to encourage physical activity among preschoolers. In general, parents were supportive of the concept as well as the initial app prototype. Although the app will likely not serve to keep children calm and quiet in a public setting, parents envisioned a variety of scenarios in which the app would be useful, ranging from breaks on long car trips to snowy days where children were stuck inside and needed an easy way to be active. A few parents expressed concern that the app would not be entertaining enough for some children, but children were engaged by it and wanted to play it several times. This may address the need to help communicate the appeal and value of the app to parents so that they choose to offer it to their children. Prototype pretesting with preschoolers indicated that the approach to the app is feasible, as children reacted positively to the app. A majority of children were up on their feet for the full length of the app, indicating that the app has the possibility to encourage activity in this age group. Furthermore, children were actively moving in a similar way during the first and second rounds of use, indicating that app has the potential to sustain children’s interest through multiple uses.

### Implications for Research and Practice

The physical activity app that was pretested as the second part of this study represents an innovative way to tap into parents’ desire for balance in mobile device use. It fulfills parents’ desire for there to be a learning benefit for children, as they will not only be learning language related to movement but will also be practicing foundational gross motor skills. The app appears to have utility for children for both independent and co-use settings, indicating it can be easily used by families in which children primarily use apps alone and in families where co-use is more frequent. In prototype pretesting, preschoolers were entertained by the app, which would give parents a few minutes of time to get other tasks completed while providing a healthy alternative to sedentary apps. At the same time, some parents mentioned that they would engage with their children around the app. Additionally, if presented in the right way, the app could serve as inspiration and a way to launch additional parent-child interactions related to movement in real-world settings.

### Strengths and Limitations

A key strength of this study was the focus on low-income, rural families, as there is limited literature on the use of mobile devices with preschoolers in this population. Second, the telephone interview script had multiple probes and gave participants ample time to share their full values and opinions related to mobile device use in their families. A rigorous data analysis was performed, as exemplified by the phone interview transcripts being comprehensively coded in multiple rounds and then further analyzed for interpretation. Finally, prototype pretesting was conducted with both parents and children, getting valuable initial input from both groups of targeted users.

However, this study is not without limitations. Although every effort was made to encourage participation from all families enrolled at study preschools, a selection bias may have occurred as recruitment flyers indicated that the interview topic would be mobile devices. Therefore, parents who were frequent users of mobile devices may have been more likely to participate. Due to logistical constraints, the preschoolers who tested the app were a sample from a local preschool, rather than a rural preschool. It is unlikely that these preschoolers are substantially different from their rural peers in response to mobile apps, but future rounds of testing will be conducted in a rural setting with children of lower socioeconomic status to confirm this assumption. Future steps will include testing the app with parent-child dyads to assess parent-child interactions related to the app as well as exploration of individual intervention components, such as the app, on health outcomes (ie, physical activity).

### Conclusions

As mobile devices are already integrated into most families’ daily routines, a free app for study participants could serve as an innovative mode of intervention delivery. Most families in the settings that were sampled already allowed their children to play on devices every day. In the interviews, parents indicated that if they were provided with this type of educational app, it would be used as a replacement for existing activities on mobile devices, easing concerns that an intervention delivered through mobile devices would dramatically increase young children’s screen time. Although this study focused on pretesting of a physical activity app, the findings related to parents’ beliefs, values, and practices related to mobile device use, which can be applied to other health and nutrition subject areas. Thorough formative research, combined with mobile app development and prototype testing, ensures that mHealth intervention strategies are accepted by and fit into the daily lives of preschoolers and their families living in rural communities.

## Abbreviations

mHealth: mobile health
